# Metabolic reset purification program improves antioxidant balance and gut microbiome in individuals transitioning to a healthier diet

**DOI:** 10.3389/fnut.2025.1621709

**Published:** 2025-08-11

**Authors:** Chinmayee Panda, Ralph Kruse, Kaya Williams, Brea Nance, Maruti Gudavalli, Shirin Pourafshar, Brandon Metzger, Slavko Komarnytsky

**Affiliations:** ^1^Nutrition Innovation Center, Standard Process Inc., Kannapolis, NC, United States; ^2^Keiser University, College of Chiropractic Medicine, West Palm Beach, FL, United States; ^3^Plants for Human Health Institute, NC State University, Kannapolis, NC, United States; ^4^Department of Food, Bioprocessing, and Nutrition Sciences, North Carolina State University, Raleigh, NC, United States

**Keywords:** whole food, botanical, detoxification, oxidative stress, gut microbiota, metabolic health, plant-based diet, healthier lifestyle

## Abstract

A healthy diet helps transition individuals towards numerous long-term metabolic benefits but is challenging to maintain. Supplementation with additional whole food-based concentrates and herbs may support this shift by enhancing metabolic resilience. This randomized, controlled, crossover study evaluated a 21-day purification program emphasizing plant foods and whole food supplementation on metabolic detoxification, oxidative stress, and gut microbiota composition. Participants followed a plant-based diet for 7 days, with lean proteins introduced from days 8–21. Whole food supplements rich in polyphenols, glucosinolates, and prebiotic fibers were included to support metabolic and gastrointestinal function. The intervention group exhibited 9% (*p* < 0.0001) reduction in ROS-associated oxidative stress compared to diet alone, suggesting enhanced redox balance. Additionally, the intervention group exhibited higher urinary creatinine levels, indicating enhanced kidney filtration efficiency (*p* = 0.03). Self-reported stress levels decreased by 36% (*p* = 0.049) in the intervention group, with a notable reduction in anxiety related to future uncertainty, demonstrating the psychological benefits of dietary supplementation. Gut microbiome analysis revealed a reduction in *Proteobacteria*, a phylum associated with chronic metabolic and inflammatory diseases, and beneficial increases in *Ruminococcus gnavus*, *Lactobacillus zeae*, and *Roseburia faecis*, which contribute to gut barrier integrity and butyrate production. These findings highlight the potential of whole food-based supplementation to enhance dietary transitions by reducing oxidative stress, supporting kidney function, alleviating psychological stress, and modulating gut microbiota composition.

**Clinical trial registration:**

NCT05877365.

## 1 Introduction

There is a growing trend toward healthier lifestyles, with more people recognizing the benefits of balanced nutrition and regular exercise (1). Many attempt to transition to a healthier diet by reducing processed foods, cutting back on sugar, and incorporating more whole foods (2). Dietary changes are also often associated with a desire to reduce intake of naturally occurring toxins such as molds and their volatile metabolites, food allergens, pesticides, persistent organic pollutants, synthetic preservatives and colorants, volatile organic compounds from solvents and fuels, and microplastics that enter modern agricultural food chains (3). These exposures have been linked to an increased risk of obesity (4), cardiovascular disease (5), neurocognitive impairment (6), and reproductive issues (6), raising concerns about the long-term health impacts of even seemingly healthy dietary patterns.

Plant-based diets and lean protein sources are increasingly seen as healthier choices, offering benefits such as improved heart health, better digestion, and lower inflammation (7). However, the cost and accessibility of fresh, safe, and nutritious foods is often a barrier, making it harder for many individuals to maintain a healthier diet (8). The change to a healthier diet is also complicated by deeply ingrained eating habits, social influences, and the convenience of unhealthy options (9). The transition requires not just willpower but also knowledge about proper nutrition to ensure a well-balanced diet (10). Despite these challenges, the shift toward healthier eating continues to grow as more people seek long-term wellness and disease prevention.

Whole foods offer significant benefits in navigating the complexities of a healthy diet, as they provide essential nutrients without the added risks of excessive processing and artificial additives (11). Consuming whole, unprocessed foods such as fruits, vegetables, whole grains, lean proteins, and healthy fats improves intake of vitamins, minerals, and beneficial plant compounds (12). Whole foods also contain significant amounts of fiber (13) and polyphenols (14) that promote better metabolic function and enhanced gut integrity, which are crucial for long-term wellness. By focusing on minimally processed ingredients, individuals can better control their exposure to harmful substances while maximizing nutritional benefits (15). Whole food powders can be a convenient strategy to supplement beneficial plant components that regulate oxidative stress and promote beneficial shifts in the microbiome, supporting resilience against environmental and dietary stressors (16). These whole food powders can serve as targeted nutritional tools to complement whole-food diets and optimize health outcomes (17).

Supplementation with whole food powders incorporates a variety of plant-based ingredients in their natural form, preserving the full spectrum of bioactive compounds such as polyphenols and glucosinolates. By utilizing whole foods, these blends offer a more comprehensive nutritional profile, supporting antioxidant balance, gut health, and overall metabolic function (18). Examples may include grape seed extract as a rich source of polyphenols and phenolic acids (19), kale and Brussels sprouts that contain glucosinolates contributing to their detoxifying and anti-inflammatory effects (20), beetroot and apple fibers as rich sources of prebiotics and phenolic acids known for their antioxidant and anti-inflammatory benefits (21), and red clover enriched with isoflavones that improve hormone regulation and antioxidant activity (22).

Emerging wellness based dietary intervention programs focus on a diverse range of nutrient-dense foods to support both dietary needs and a healthy weight, but also support individuals maintain a healthier diet and lifestyle after the program is completed (23). This approach shifts the focus from long-term restrictive dieting to a more inclusive, sustainable mindset, encouraging a gradual switch to positive lifestyle habits rather than guilt-driven food avoidance (24). In this study, we focused on a 21-day purification program that emphasizes whole foods while temporarily eliminating potential dietary triggers. Participants consumed a variety of vegetables and fruits for the first 7 days, with select lean proteins introduced on days 8–21 to support balanced nutrition. Whole food-based powders were integral to the program, promoting overall wellness and supporting the transition to a healthier diet during and after the program is completed. The primary objective was to evaluate functional markers of metabolic detoxification in urine. Secondary outcomes were to determine oxidative stress, serum biomarkers, self-reported wellness data, and short-term shifts in the gastrointestinal microbiome profiles associated with whole food supplementation and transition to a healthier diet.

## 2 Materials and methods

### 2.1 Trial design

**Figure 1:**
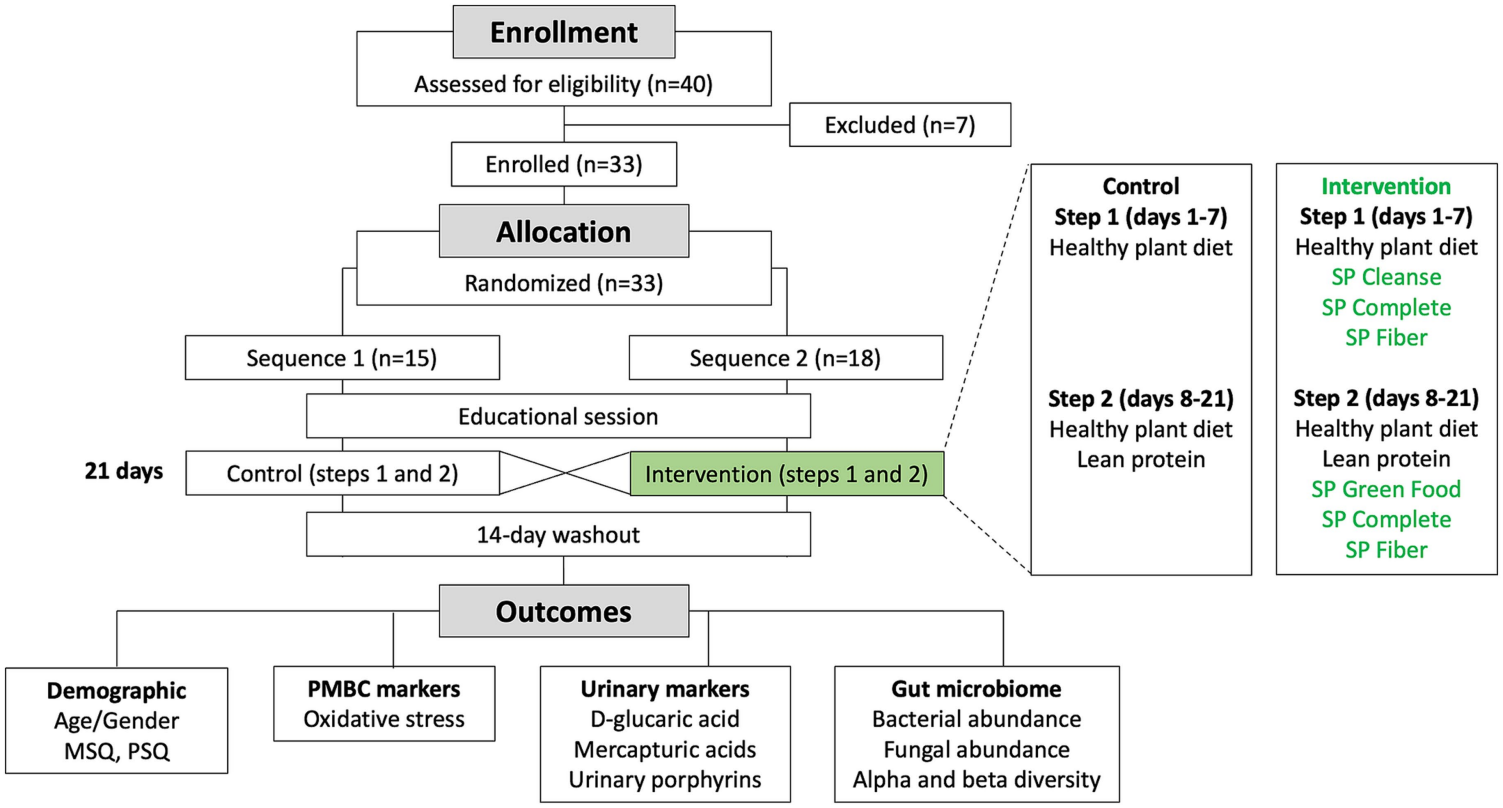
Flowchart of the study.

The study was a randomized, double-blind, two-sequence crossover trial (RCT). Flow of the participants through the study is shown in Figure 1. Each sequence consisted of 2 steps: a transition to a plant diet on days 1–7 (step 1), followed by a plant diet with added lean protein on days 8–21 (step 2). The control condition included a dietary educational session and a transition to a new diet only. The intervention condition included a dietary educational session and a transition to a new diet, supported by daily supplementation with Standard Process (SP) Cleanse, SP Complete, and SP Fiber during days 1–7 (step 1), and SP Green Food, SP Complete, and SP Fiber during days 8–21 (step 2). Each participant underwent both the control condition and the intervention condition in the random order, with a 2-week washout period in between. *A priori* power analysis was conducted, assuming an attrition rate of approximately 20% and a within-subject (pre-post) comparison design, a sample size of approximately 20 participants was calculated to provide 90% power (*α* = 0.05) to detect a standardized effect size (Cohen’s d) of 0.8 (large effect). The study was initiated in July 2023 and completed in October 2023 at Keiser University Spine Care Clinic in West Palm Beach, Florida.

### 2.2 Participants

Study subjects were heathy adults (> 18 years of age) who expressed the wish to transition to a healthier lifestyle with intention to improve their emotional, mental, and/or physical health and wellbeing. After successfully completing the enrollment questionnaires, meeting eligibility criteria and indicating interest in joining the study, subjects received the subject information sheet and signed the informed consent form. All eligible subjects were randomized using a stratified block randomization approach with an allocation ratio of 1:1 to one of two sequences. Overall, 40 potential participants responded to a posting, and 33 were subsequently offered participation in the program, resulting in 15 and 18 individuals per sequence. Subjects and study staff were blinded as to study condition and randomization sequence.

There were no protocol deviations at the end of the study. Additional dropouts from the study were evenly distributed between the study groups and included an undisclosed medical condition (1), inability to follow the study protocol, moving away from the study area (1), getting pregnant (1), and no reason given (6). Participant adherence to the dietary and supplementation protocols was monitored via a daily intake journal to record their food log, supplements intake, and physical activity. Participant information and generated data were fully anonymized for data analysis and interpretation of results. All research involving human participants was approved by the Advarra Institutional Review Board (protocol no Pro00062082, approved on March 31, 2022) and all clinical investigation was conducted according to the principles expressed in the Declaration of Helsinki. Informed consent was obtained from all subjects involved in the study. The study has been registered on www.clinicaltrials.gov (Identifier: NCT05877365).

### 2.3 Inclusion and exclusion criteria

Study inclusion criteria were: healthy adults (> 18 years of age) in college settings who are willing to make lifestyle changes; willing to sign and date an informed consent form; willing to comply with the study protocol for 58 days; willing to come and provide samples on all 4 study visits; no allergy to any study products; a male or a non-pregnant, non-lactating female, at least 6 weeks postpartum prior to screening visit, and is not ac-tively planning a pregnancy; if on a chronic medication (that does not result in exclusion), on stable dose for at least 2 months prior to screening visit; willing to stay compliant and not participate in another research study.

Study exclusion criteria were: prohibited medications, supplements or herbal products; any adverse events due to any nutraceutical, OTC, or pharmaceutical or in-vestigational products; celiac and other gastrointestinal health concerns; the use of lipid lowering drugs or anticoagulant medications in the preceding 4 weeks and for duration of study; pregnant and nursing women, and women of childbearing age expecting to be pregnant soon; untreated endocrine, neurological, or infectious disease; the diagnosis of HIV disease or AIDS; significant liver or kidney disease; rheumatoid arthritis, ankylosing spondylitis, systemic lupus erythematosus, polymyositis, scleroderma, polymyalgia rheumatic, temporal arteritis or Reiter’s syndrome; psoriasis, deep vein thrombosis or pulmonary embolus; history of cancer; high triglyceride levels >150 mg/dL; use of ethanol within 24 h of the evaluation visits; any other sound medical, psychiatric and/or social reason as determined by the study director; co-enrollment in other studies was restricted.

### 2.4 Educational session

The healthy diet education session included a PowerPoint presentation and a matching handout with dietary guidelines, as well as a sample list and a sample meal plan of nutrient-rich plant foods including vegetables, fruits, legumes, pseudo-grains, oils, fats, and lean meats (fish, red meats, poultry, and wild game). The subjects were asked to refrain from consuming or using alcohol, caffeine, tobacco or other stimulants, dairy, eggs, grains, nuts, processed or refined foods, shellfish, and soy products. Participants were also educated on continuing to follow a plant diet with lean protein included after study completion, as well as provided with general guidelines on reintroducing foods eliminated during the study. The intervention component of the educational program included an additional PowerPoint presentation with the information about the investigational products, directions, and dosing information for their consumption.

### 2.5 Study investigational products

The SP Cleanse is a whole-food blend supplied by the manufacturer (Standard Process Inc., Palmyra, WI, USA) as 400 mg capsules with juniper berry powder, organic red clover (aerial parts), collinsonia (root), apple pectin, burdock (root) powder, organic barley (grass), dandelion (leaf), organic beet (root), organic Spanish black radish (root), Oregon grape (root) powder, organic cayenne pepper (*Capsicum annuum*), fenugreek (seed), inositol, globe artichoke (leaf), fennel (seed), milk thistle extract (80% silymarins), organic cordyceps mushroom powder, broccoli (aerial parts), organic kale (aerial parts), organic carrot, and organic sweet potato. The serving size was 7 capsules, 3 times per day on an empty stomach or as directed (step 1, days 1–7 only).

SP Complete is a whole-food blend supplied by the manufacturer (Standard Process) as 795 g bottles with whey protein, flax meal, rice protein, calcium citrate, magnesium citrate, organic buckwheat (aerial parts), organic Brussel sprouts (aerial parts), organic kale (aerial parts), inositol, organic alfalfa (aerial parts) juice powder, sunflower lecithin powder, grape seed extract, and organic carrot. The serving size was 2 rounded tablespoons (25 g), 2 times per day (steps 1–2).

SP Whole Food Fiber is a whole-food blend supplied by the manufacturer (Standard Process) as 200 g bottles with oat fiber, beet fiber, rice (bran), organic beet (root), apple pectin, organic carrot (root), organic sweet potato, and carrot fiber. The serving size was 1 tablespoon (6 g), 3 times per day (steps 1–2).

SP Green Food is a whole-food blend supplied by the manufacturer (Standard Process) as 800 mg capsules with organic Brussel sprouts (aerial parts), organic kale (aerial parts), organic buckwheat (aerial parts), organic barley (aerial parts) juice powder, organic alfalfa (aerial parts) juice powder, and organic buckwheat (aerial parts) juice powder. The serving size was 5 capsules, 2 times per day (step 2, days 8–21 only).

### 2.6 Demographic information

Baseline measures (age/gender) were recorded by the study staff at the time of consent. The standard Medical Symptoms Questionnaire (MSQ) was administered to all participants to monitor study readiness and health-related events during the study.

### 2.7 Laboratory testing

**Figure 2:**
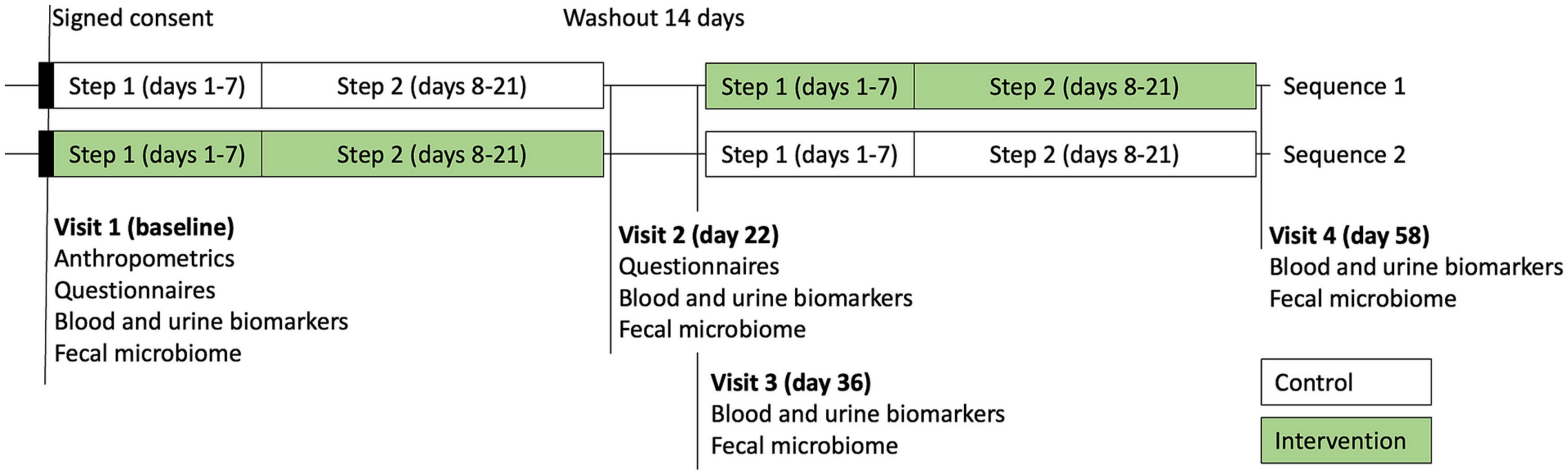
Study visits and sample collection timepoints.

Morning first void urine samples at each of 4 visits were collected for assessment of the hepatic detoxification profile (phase I metabolite D-glucaric acid, phase II metabolite mercapturic acids, and creatinine) and the total urine porphyrins using the commercial testing services (Doctor’s Data, St. Charles, IL) (Figure 2). Fasting blood samples were collected into three BD Vacutainer SST tubes, allowed to clot at room temperature for 30–45 min, then serum was separated by centrifugation at 3,400 rpm for 10 min, and stored at −80°C. Urinary detoxification biomarkers were independently collected and analyzed by Doctor’s Data (St. Charles, IL), a clinical laboratory testing service not affiliated with the sponsor.

### 2.8 Oxidative stress

Blood samples were collected in BD Vacutainer CPT mononuclear cell preparation tubes that contained blood separation media composed of a thixotropic polyester gel and a Ficoll Hypaque solution. The tubes were centrifuged to isolate live peripheral blood mononuclear cells (PBMC), and oxidative stress was measured using a fluorogenic cell-permeant probe CellROX Orange (Thermo Fisher, Waltham, MA). Fluorescence upon oxidation by reactive oxygen species was quantified on BD Accuri C6 flow cytometer with absorption/emission maxima of 545/565 nm and presented as relative fluorescent units (RFUs).

### 2.9 Self-reported wellness

The participants reported self-perception of stress using the Perceived Stress Questionnaire (PSQ). The questionnaire queries stressful feelings and experiences over the course of the last month on the scale 1 (almost never), 2 (sometimes), 3 (often), and 4 (usually). A total score was calculated as a sum of 30 values, including positive questions 1, 7, 10, 13, 17, 21, 25, and 29 converted to 5-score values. The PSQ index was calculated by subtracting 30 from the raw score and dividing by 90 (25).

### 2.10 Fecal sample collection and DNA amplification

Fecal samples from participants were collected using a sterile BD BBL CultureSwab EZ (Franklin Lakes, NJ, USA) and, immediately submerged in 1 mL of InhibitEX Buffer (Qiagen, Hilden, Germany), and frozen until further use. Fecal swabs in the lysis buffer were thawed and incubated for 1 h at 75°C. Samples were then shaken twice using Fastprep 96 (MP Biomedicals, Solon, OH, USA) for 5 min at 1,800 rpm before centrif-ugation woth Eppendorf Centrifuge 5,810 (Hamburg, Germany) at 4,000 rpm for 5 min. Equal amounts of 100% ethanol and lysate were mixed in a sterile 96-well plate and then transferred to Wizard SV 96 Binding Plate (Promega, Madison, WI, USA). Samples were passed through using a vacuum manifold. Residual contaminants were removed by subsequent washing with 70% ethanol with 0.05% Tween 20 (Fisher Scientific) (Wash 1) and 70% ethanol (Wash 2), before eluting DNA with 80 μL of molecular biology-grade water. The purity and quality of the isolated genomic DNA was confirmed and quan-titated with the NanoDrop OneC (Thermo Scientific, Waltham, MA, USA) applying the dsDNA Assay.

Amplifications of the V3-V4 regions of 16S and ITS1 region of the 5.8S rRNA genes were performed using 16S-515 (5′-GGA CTA CCA GGG TAT CTA ATC CTG- 3′) and 16S-804 (5’-TCC TAC GGG AGG CAG CAG T-3′) and ITS1 (5’-TCC GTA GGT GAA CCT GCG G- 3′) and ITS4 (5’-TCC TCC GCT TAT TGA TAT GC- 3′) primers, respectively. The PCR mixture was composed of GoTaq HotStart Green Master Mix (Promega, Madison, WI, USA) at a 1x and 0.05 μL/mM of each primer. Undiluted DNA (1.5 μL) was added to each 50 μL reaction. Thermo-cycling conditions consisted of an initial denaturation step (3 min at 98°C), followed by 30 cycles of denaturation (10 s at 98°C), annealing (10 s at 55°C for the 16S primers and 20 s at 58°C for the ITS primers), extension (10 s at 72°C), and a final extension step of 3 min at 72°C. To confirm amplification, the PCR products (10 μL) were separated using gel electrophoresis on 1.5% agarose gel.

### 2.11 Microbiome sequencing and analysis

Microbiome sequencing and analysis was performed independently by BIOHM Health (Cleveland, OH), a commercial microbiome testing service not affiliated with the sponsor. The sequencing library for next-generation sequencing was prepared following the Ion Torrent workflow according to the manufacturer’s instructions (Thermo Fisher Scientific, Waltham, MA, USA). Equal volumes of bacterial 16S and fungal ITS amplicons were pooled, cleaned with Sera-Mag Select magnetic beads (Cytiva Life Sciences, Marlborough, MA, USA) to remove unused primers, and then exposed to end repair enzymes for 20 min at room temperature. Ligation was then performed at 25°C for 30 min using Ion Torrent P1 and a unique barcoded ‘A’ adaptor per pooled sample. After magnetic bead removal of residual adaptors, all separate barcoded samples were then pooled in equal amounts (10 μL) and size selected for the anticipated 16S and ITS range (200–800 bp) using Pippin Prep (Sage Bioscience, Beverly, MA, USA). The sequencing library was then amplified for seven cycles and quantitated on StepOne qPCR instrument (Applied Biosystems, Waltham, MA, USA) ahead of proper dilution to 300 pM going into IonSphere templating reaction on the Ion Chef system (Thermo Fisher Scientific, Waltham, MA, USA). Library sequencing was completed on an Ion Torrent S5 sequencer (Thermo Fisher Scientific, Waltham, MA) and barcode-sorted samples analyzed based on Greengenes V13.8 and Unite database V7.2 designed for the taxonomic classification of 16S rRNA and ITS sequences, respectively.

Alpha and beta diversity were calculated using the phyloseq package in R (26). Significance in alpha diversity difference were checked using the package SplinectomeR (27). NBZIMM3 was used to identify significant changes over time by modeling taxa abundance using a negative binomial mixed model, accounting for longitudinal correlations and covariates (28).

### 2.12 Data analysis

The subject data was collected and analyzed at the Keiser University Spine Care Clinic (West Palm Beach, FL), a clinical site not affiliated with the sponsor. Blood biomarkers of oxidative stress were collected and analyzed at the NC State University (Kannapolis, NC), an academic lab not affiliated with the sponsor. Urinary detoxification biomarkers were independently collected and analyzed by Doctor’s Data (St. Charles, IL), a clinical laboratory testing service not affiliated with the sponsor. Finally, microbiome sequencing and analysis was performed independently by BIOHM Health (Cleveland, OH), a commercial microbiome testing service not affiliated with the sponsor.

Statistical analyses were performed using JMP 15.2.1 (SAS Institute, Cary, NC) and Prism 8.0 (GraphPad Software, San Diego, CA, USA). Descriptive statistics and two-way repeated measures ANOVA were used to evaluate changes in clinical outcomes at baseline and after intervention. Data was analyzed twice, first by Intention to Treat analysis (all observations), then by Per Protocol analysis (excluding all dropouts). Values are reported as mean +/− standard deviation (SD), and statistical significance was set at *p* ≤ 0.05. Asterisks *, **, ***, **** indicate significance levels of *p* < 0.05, *p* < 0.01, *p* < 0.001, and *p* < 0.0001, respectively.

## 3 Results

### 3.1 Subjects characteristics

A total of 33 healthy participants (25 female, 8 male, age range 21–64 years) were selected for the study and randomized into two crossover study sequences (control/intervention, sequence 1) or (intervention/control, sequence 2) (Figure 1). Seven additional participants were screened but excluded from participating in the study because they were unable to commit to the study protocol. Demographics data of participants are shown in Table 1.

**Table 1:** Demographic data of the study participants (± SEM).

Characteristics	Sequence 1 (*n* = 15)	Sequence 2 (*n* = 18)
Age (years)	32.3 ± 11.7	28.9 ± 7.5
Gender (male/female, %)	2/13 (13.3%)	6/12 (33.3%)

Both sequences received an educational session during visit 1 of the study that included information and a handout with healthy dietary guidelines to support the participants while transitioning to a healthier plant-based diet (Figure 2). A sample list and a meal plan of the suggested healthier nutrient-rich plant foods was provided to be used as a guide during step 1 (days 1–7); a sample list and a meal plan of the suggested healthier lean meats was provided to be used as a guide during the step 2 (days 8–21). The intervention component of the educational program included additional information about the investigational products, directions, and dosing requirements for their consumption.

### 3.2 Biomarkers of oxidative stress

**Figure 3:**
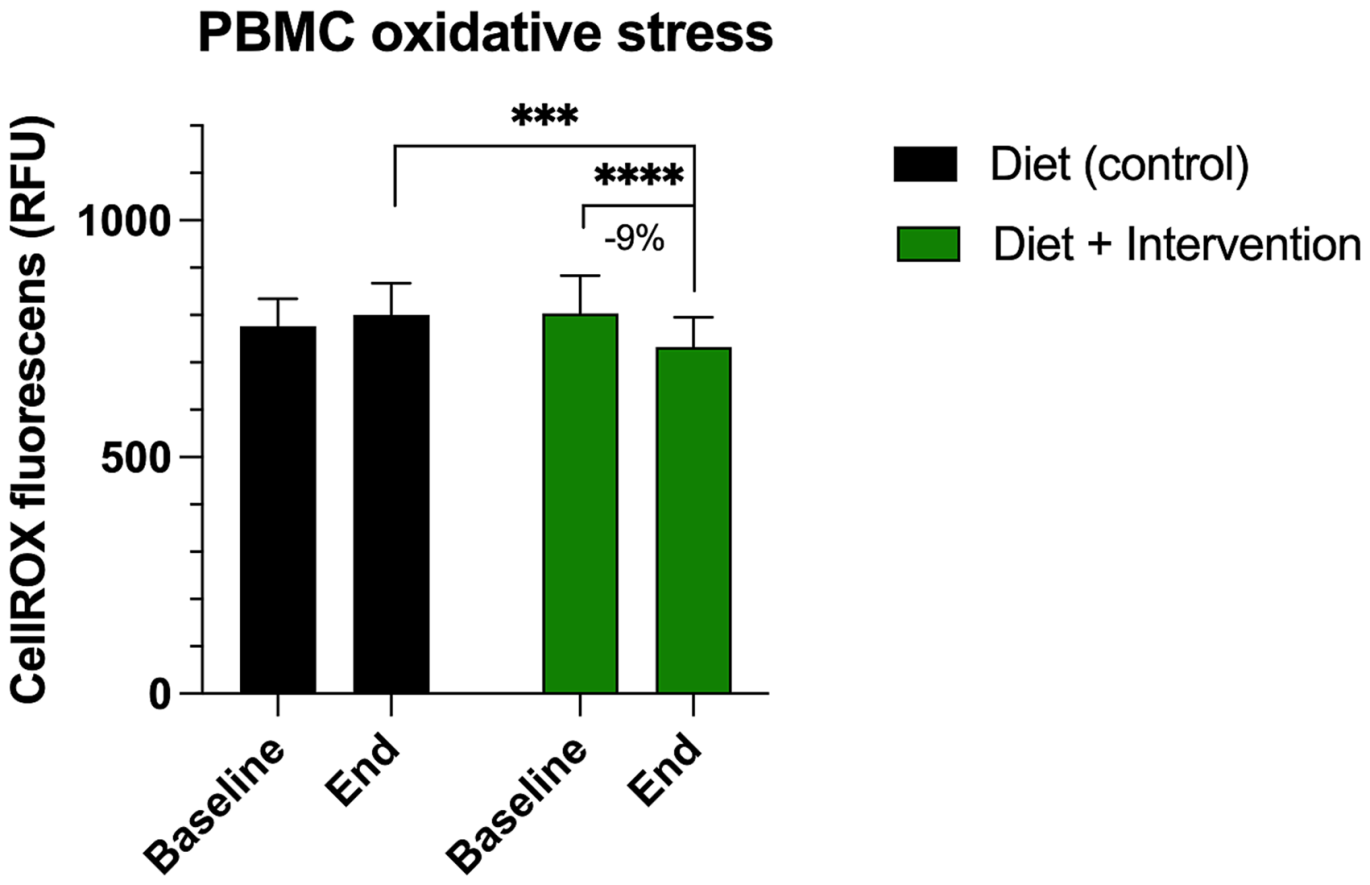
Changes in the levels of oxidative stress in the peripheral blood mononuclear cells (PBMC) measured by CellROX orange reagent. Results were expressed as means ± SD. Data were analyzed by the two-way ANOVA followed by Fischer’s LSD test (***p < 0.001, ****p < 0.0001).

At the baseline, the ROS-associated oxidative stress in the peripheral blood mononuclear cells was observed at 776.8 ± 58.1 RFU for the control group (diet alone) and 802.7 ± 79.3 RFU for the intervention group (diet and dietary supplementation). At the end of the study, we observed a 9% decrease in PMBC oxidative stress only in the participants receiving diet and dietary supplementation (731.2 ± 62.8 RFU, *p* < 0.0001) (Figure 3). This value was also significantly lower than the end of the study oxidative stress value observed in the control group (800.1 ± 67.6 RFU, *p* = 0.0006).

### 3.3 Detoxification markers in urine

Consistent with previous reports of high interindividual variability in detoxification panels, we observed no significant reductions in total urinary porphyrin biomarkers in either the control or intervention cohorts (all *p*-values > 0.05, Table 2). There were also no changes in the urinary secretion of D-glucaric acid, indicating stable phase I detoxification activity in all groups. Transition to the plant-based diet alone (control group) resulted in significant elevation of urinary mercapturic acids (46.5 ± 20.0 to 62.6 ± 35.3 μM/mM, *p* = 0.007), a biomarker of increased phase II detoxification and glutathione conjugation. This effect was not observed in the intervention group (Table 2). Finally, increased urinary creatinine was observed only in the intervention group that could indicate enhanced kidney filtration efficiency (134.6 ± 53.6 to 171.9 ± 81.6, *p* = 0.039), possibly influenced by whole food supplementation, but not the overall dietary change.

**Table 2:** Hepatic detoxification markers in urine (± SD).

Variable	Diet (Control)			Diet + Intervention			Reference range
	Baseline	End of study	*p*	Baseline	End of study	*p*	
Total porphyrins, nmol/L	104.8 ± 33.0	104.7 ± 36.6	0.98	105.7 ± 28.3	99.4 ± 27.7	0.45	< 320
D-glucaric acid, nM/mg	65.1 ± 57.5	51.6 ± 30.1	0.23	44.8 ± 23.7	59.8 ± 54.7	0.24	40–400
Mercapturic acids, μM/mM	46.5 ± 20.0	62.6 ± 35.3**	0.007	46.6 ± 18.5	57.9 ± 12.5	0.09	40–95
Creatinine, mg/dL	118.4 ± 57.2	116.0 ± 51.8	0.88	134.6 ± 53.6	171.9 ± 81.6 *	0.039	40–325

Data were analyzed by the two-way ANOVA followed by Fischer’s LSD test (*p < 0.05, **p < 0.01).

### 3.4 Wellness status and perception of stress

No adverse effects attributable to the intervention were reported during the study. There were also no significant observations in the MSQ questionnaire completed during the study, suggesting that all participants followed the dietary education instructions provided to them at baseline, and experienced no undesirable events during the trial.

**Figure 4:**
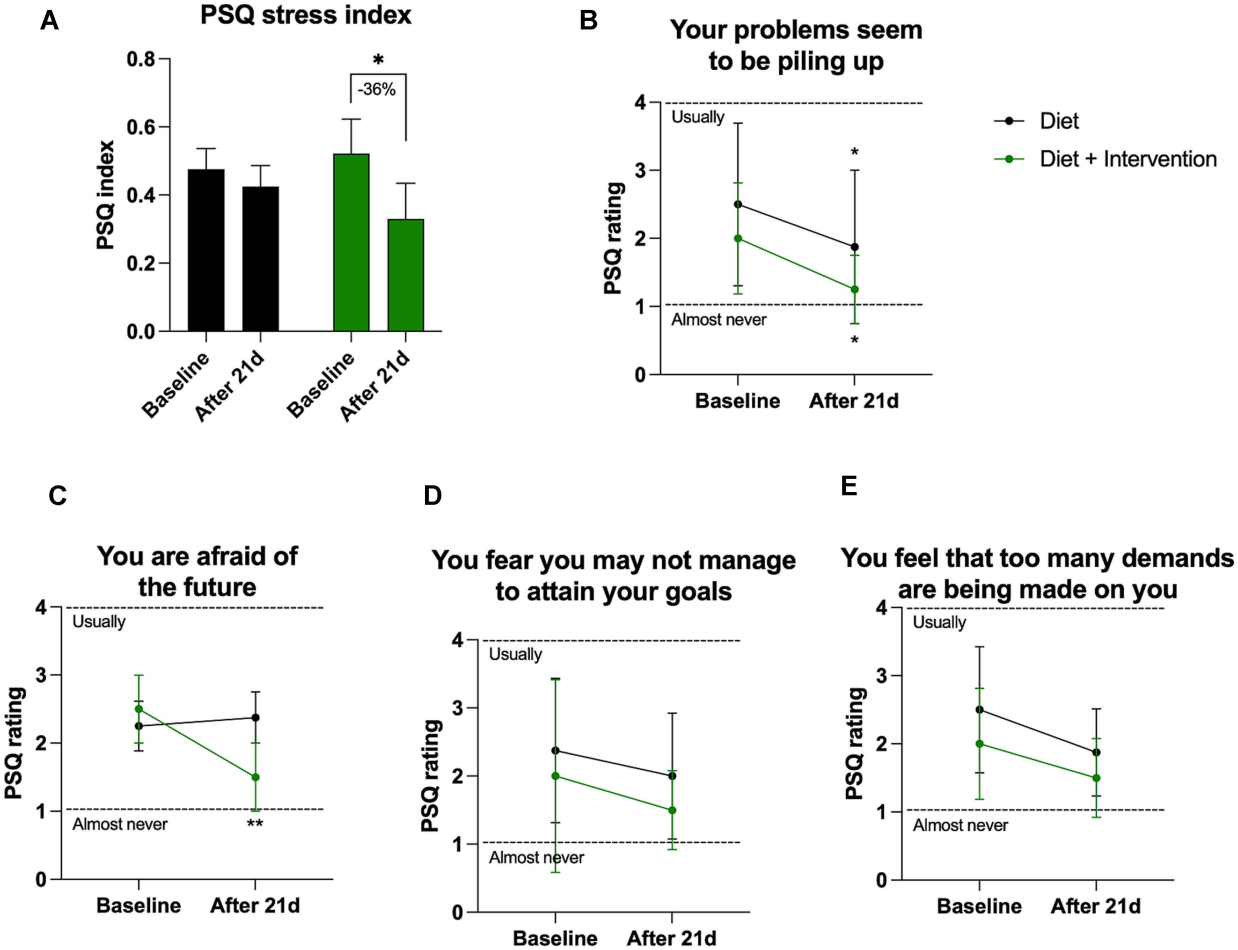
Self-reported PSQ stress perception. **(A)** Changes in the overall stress as quantified by PSQ stress index varying from 0 (lowest) to 1 (highest) level of perceived stress. Individual changes in the PSQ ratings **(B)** question 15, **(C)** question 22, **(D)** question 9, **(E)** question 2. Results were expressed as means ± SD. Data were analyzed by the two-way ANOVA followed by Fischer’s LSD test (*p < 0.05, **p < 0.01).

Mean PSQ index scores were used to evaluate self-perception of stress at the baseline and the end of the purification program. There were no differences in PSQ scores between the control and intervention cohorts at the beginning of the study, and a significant decrease in the PSQ score was observed only in the group that underwent dietary supplementation. In this group, the intervention reduced PSQ stress index by −36% (from 0.52 at baseline to 0.33 after completion of the purification program, *p* = 0.049) (Figure 4A). The largest significant reduction observed in both control and intervention cohorts were self-perception of “problems that seem to pile up” (−0.64 PSQ rating points in controls and −0.75 points in the intervention cohort, *p* > 0.05) (Figure 4B). The intervention was uniquely associated with a significant drop in “fear of the future” (−1 PSQ rating points, *p* = 0.004) that was absent in the control group (Figure 4C). Two other areas of reduced stress were observed in both groups in fear of “not attaining your goals” (Figure 4D) and “too many demands” (Figure 4E).

### 3.5 Changes in gut microbial community composition

**Figure 5:**
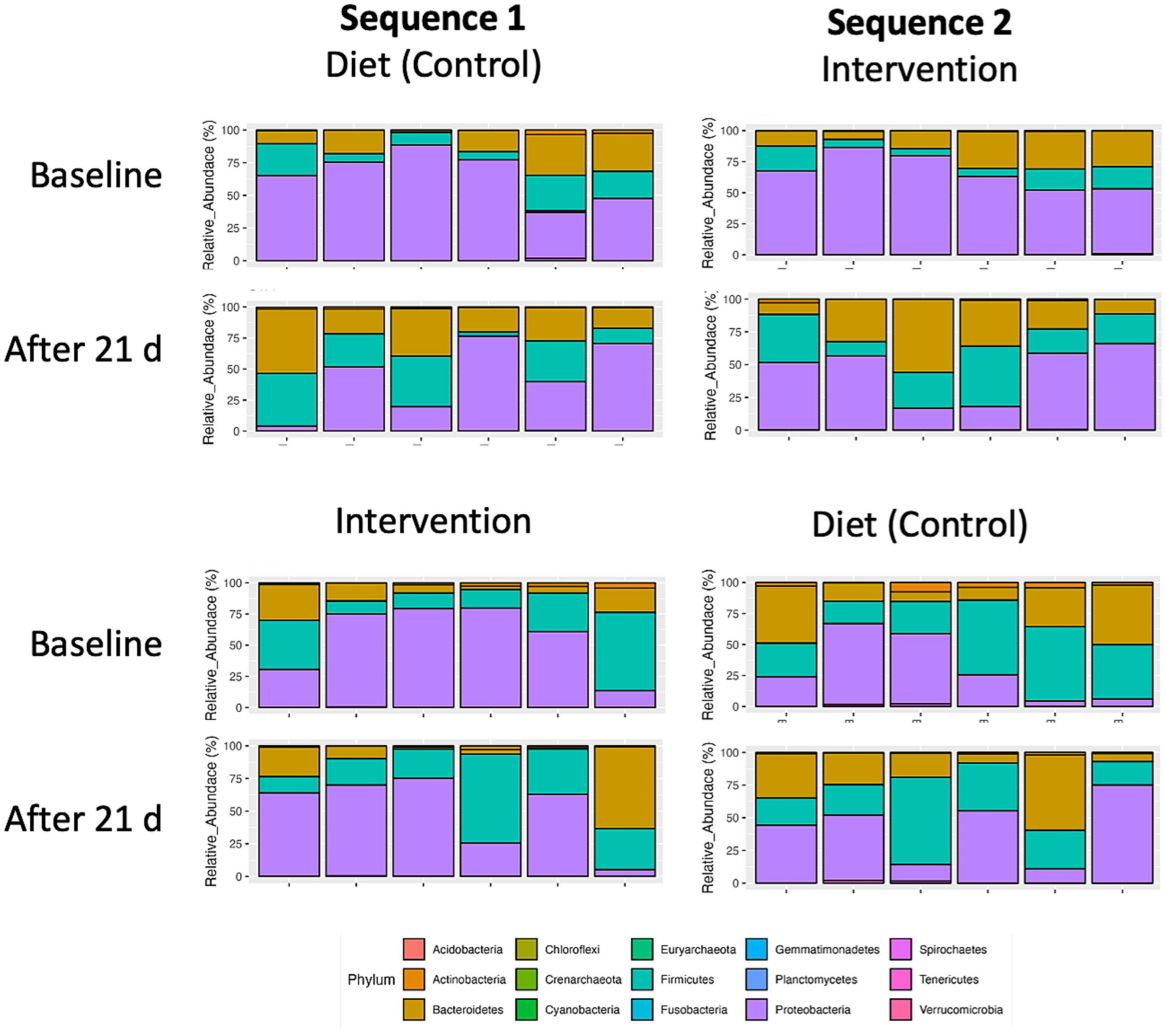
Taxonomic distribution of top 15 bacterial phyla in fecal microbiomes of individual participants (*n* = 12) undergoing either dietary transition (control) or dietary transition with dietary supplementation (intervention). Individual crossover data is separated by sequence 1 (control-intervention) and sequence 2 (intervention-control).

The global microbiome composition (*n* = 12) at baseline and each of the 4 visits both in sequence 1 (control-intervention) and sequence 2 (intervention-control) are shown in Figure 5. Additionally, the relative abundance of fecal bacteria and fungi is also presented at the phylum, genus, and species levels (Supplementary Figures S1–S6).

**Figure 6:**
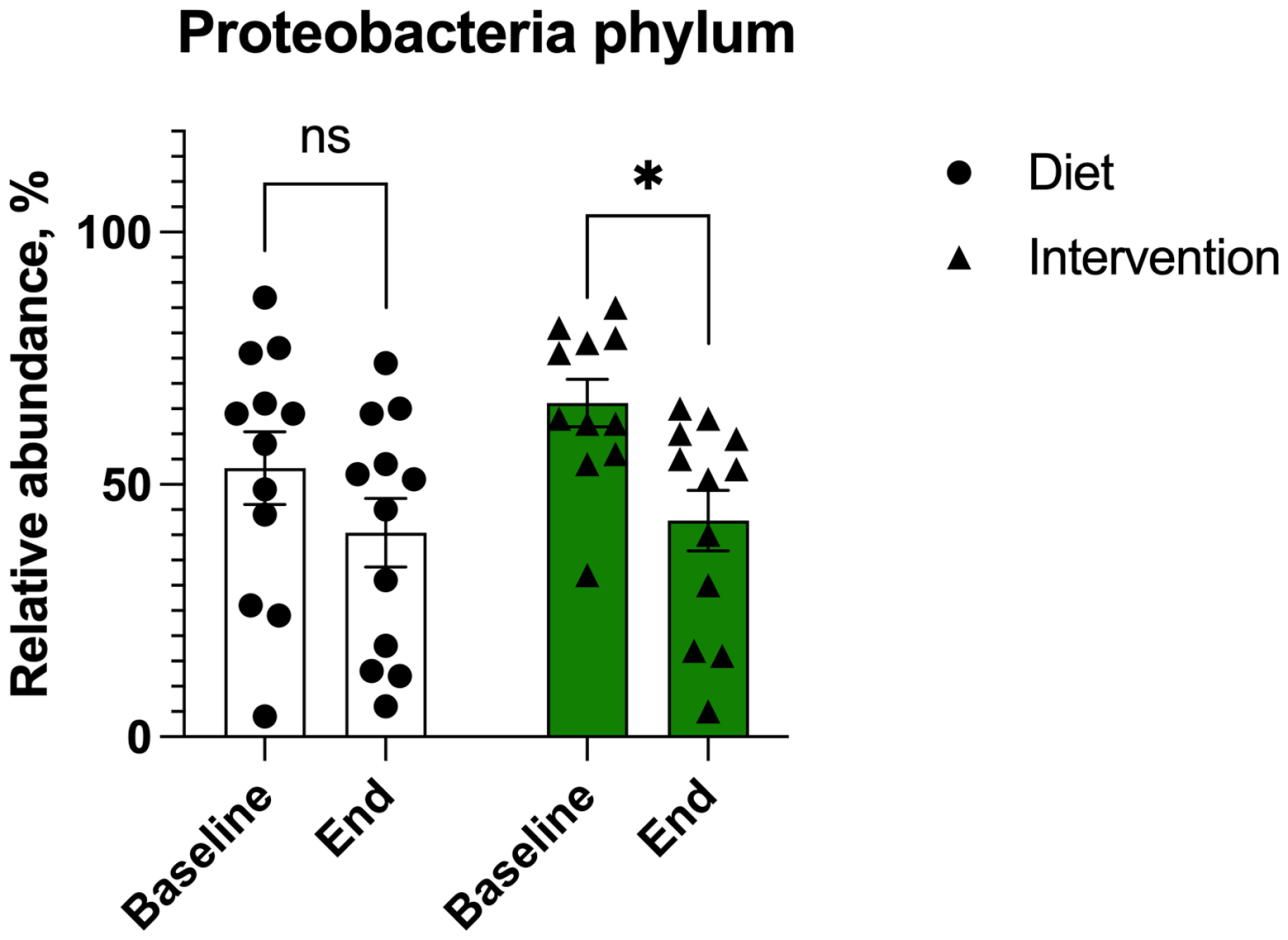
Changes in *Proteobacteria* abundance in individuals (*n* = 12) undergoing either dietary transition (diet) or dietary transition with supplementation (intervention). Results were expressed as means ± SEM. Data were analyzed by the two-way ANOVA followed by Fischer’s LSD test.

At baseline, most of participants presented with microbiome profiles characterized by high levels of *Proteobacteria*. Both the plant diet alone and the diet supplemented with intervention decreased *Proteobacteria* abundance by expanding *Bacteroidetes* and *Firmicutes* phyla. This trend was significant for the intervention (*p* = 0.056) but did not reach significance for the diet alone (*p* = 0.291) (Figure 6).

**Figure 7:**
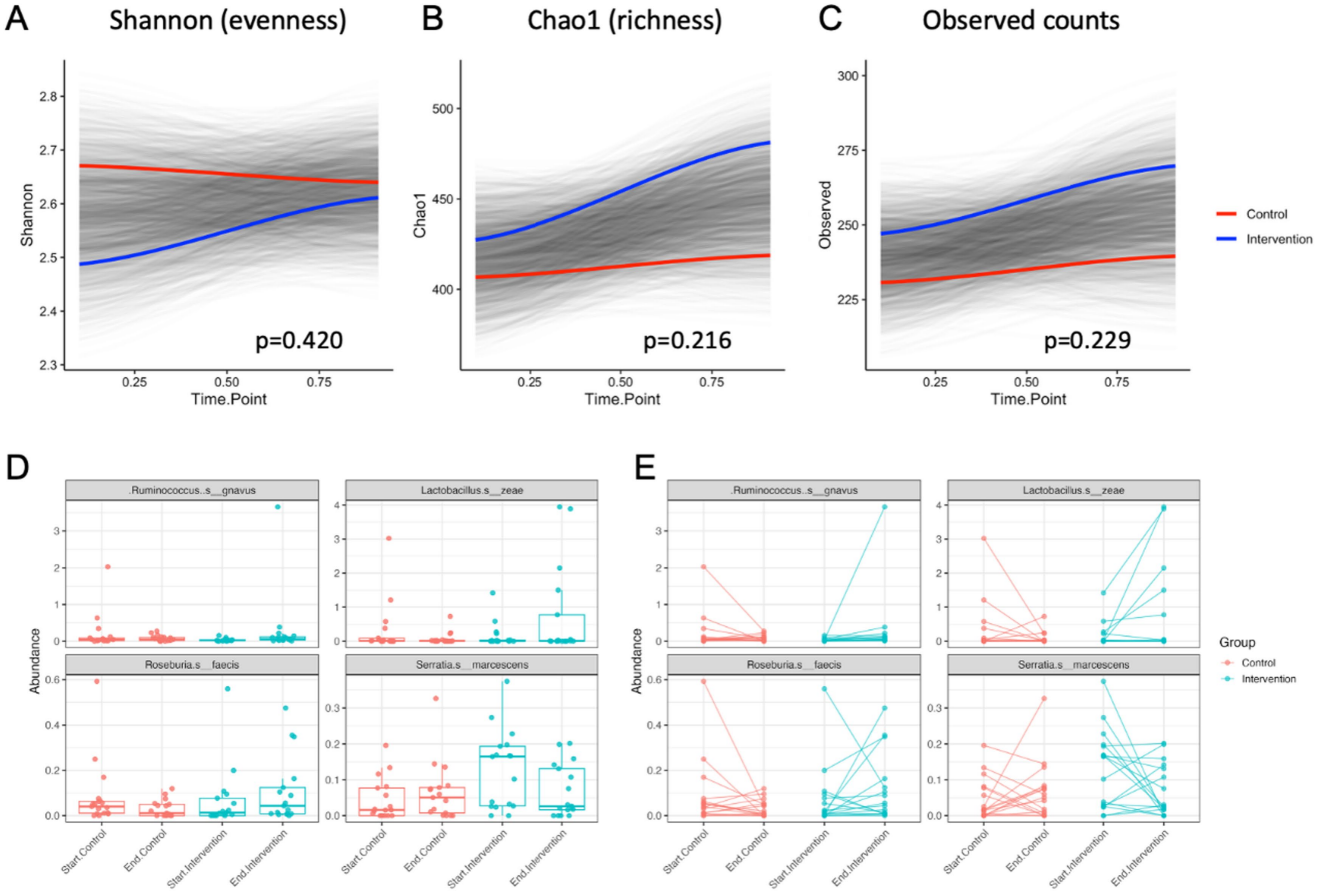
Alpha diversity values over time for control and intervention groups estimated by **(A)** Shannon index, **(B)** Chao1 index, and **(C)** the observed species counts. Most significant changes in abundance of bacterial species shown as **(D)** average means and **(E)** individual abundance shifts among the participants undergoing the control (transition to a healthy diet) or intervention (diet with supplementation) treatments.

At the end of 21-day purification program, alpha diversity calculated as Shannon index that estimates the number of taxa and how evenly they are distributed across the fecal microbiome showed no significant differences in both control and intervention samples compared to the baseline (*p* = 0.420) (Figure 7A). The Chao1 index that estimates the total species richness including rare species showed a moderate increase in the intervention group only (*p* = 0.216) (Figure 7B), as did the observed counts (*p* = 0.229) (Figure 7C). Principal coordinate analysis based on Bray-Curtis was analyzed to obtain beta diversity of the samples and showed no clustering at baseline or the end of intervention (Supplementary Figure S6).

At the species level, differential abundance in the intervention group most significantly decreased for *Serratia marcescens* (*p* < 0.05) and increased for *Ruminicoccus gnavus* (*p* < 0.01), *Lactobacillus zeae* (*p* < 0.01), and *Roseburia faecis* (*p* < 0.05) when compared to the controls (Figures 7D,E).

## 4 Discussion

The growing trend toward healthier lifestyles reflects a broader awareness of the importance of nutrition in long-term wellness. Many individuals strive to adopt a healthier diet not only due to concerns about physical health (29) and metabolic balance (30), but also because of perceived negative effects of modern agricultural practices (8), exposure to ultra-processed foods (31), environmental contaminants (32), and sustainability challenges (33). While many individuals strive to improve their diets, the transition is often met with obstacles and the challenge of changing ingrained eating habits. Whole foods and whole food powders provide a valuable approach to improving health by delivering essential nutrients without excessive processing, as well as supplementing key phytochemicals that regulate oxidative stress and support microbiome balance. These supplements can enhance the benefits of a whole-food diet, making it easier for individuals to navigate the challenges associated with healthier eating and shift the individual microbiome profiles towards better digestion of a new diet.

This randomized, controlled, cross-over study showed that dietary supplementation alongside a plant-based diet reduced oxidative stress in peripheral blood mononuclear cells, as evidenced by a 9% decrease in ROS-associated oxidative stress in the intervention group. This reduction was significant compared to both baseline levels and the control group, which followed diet alone. These findings were in line with previously reported 13% decrease in PMBC ROS observed with one of the similar botanical products after 28 days of supplementation (16). Intracellular ROS play an important role in immune cellular function and promote local and systemic inflammatory responses (34). This is especially evident in redox-sensitive signaling in inflammatory T cells (35), and may provide a better measure of oxidative stress than biomarkers of macromolecule damage (36).

At the same time, no significant changes were observed in total urinary porphyrin biomarkers or D-glucaric acid secretion, indicating stable phase I detoxification activity across all groups. The control group, following a plant-based diet alone, showed a significant increase in urinary mercapturic acids, suggesting enhanced phase II detoxification through glutathione conjugation (37). In contrast, this effect was not observed in the intervention group. The current supplementation strategy shared several whole food functional ingredients (broccoli, Brussel sprouts, kale, alfalfa, barley, juniper, and buckwheat) with the previous evaluation (16); however, the target population was younger. It is possible that in younger healthy individuals phase II detoxification or compensatory downregulation were more efficient at baseline (38) that resulted in stable phase II detoxification activity for the duration of the study.

Additionally, increased urinary creatinine levels in the intervention group suggested improved kidney filtration efficiency, potentially influenced by whole food supplementation rather than dietary change alone. There were two functional ingredients in the current supplementation strategy that could be responsible for these effects. Beets rich in betalains and nitrates (39), as well as dandelion leaf with direct diuretic properties (40) are known to enhance kidney filtration efficiency.

Self-perceived stress levels, assessed through the PSQ index, were analyzed at both baseline and the end of the purification program, and the intervention group experienced a 36% reduction in stress. Notably, the intervention group demonstrated a significant reduction in “fear of the future,” an effect that was absent in the controls. These findings suggest that dietary supplementation during purification may have a beneficial impact on stress perception, particularly in alleviating future-related anxiety. Given that stress and uncertainty are major barriers to dietary change (41), these results highlight the potential of structured dietary interventions to support individuals transitioning to a healthier lifestyle by reducing psychological stress and promoting adherence to new eating habits.

The intervention led to significant shifts in gut microbiota composition, highlighting potential benefits for host health. Notably, transitioning to a plant-based diet, followed by the addition of lean protein, resulted in a positive trend of decreased *Proteobacteria* that was significantly potentiated by supplementation. Proteobacteria are a diverse phylum of Gram-negative bacteria that include many opportunistic pathogens (42). While they are a natural part of the human gut microbiome, an increased abundance is often associated with dysbiosis, inflammation, and metabolic disorders (43). High levels of *Proteobacteria* are linked to conditions such as inflammatory bowel disease (IBD), obesity, and insulin resistance due to their potential to produce endotoxins that trigger immune responses (44). Thus, modulating *Proteobacteria* presence in the microbiome is a promising strategy for preventing and managing chronic metabolic and inflammatory diseases.

The intervention was also associated with changes in relative abundance of four bacterial species. Abundance of *Serratia marcescens* decreased, which is beneficial given its association with opportunistic infections and gut dysbiosis (45). Meanwhile, *Ruminococcus gnavus*, *Lactobacillus zeae*, and *Roseburia faecis* increased significantly, suggesting improvements in gut function. *Ruminococcus gnavus* has been linked to mucin degradation and short-chain fatty acid production, which can enhance gut barrier integrity and immune modulation (46). The rise in *Lactobacillus zeae*, a probiotic species, may contribute to improved digestion, antimicrobial activity, and enhanced metabolic health (47). Similarly, *Roseburia faecis* is known for its role in butyrate production, a key metabolite in maintaining colon health and reducing inflammation (48). These microbial shifts suggest that the intervention may promote a more favorable gut environment, supporting metabolic and immune functions in participants that are transitioning to a healthier diet.

Although the study was sufficiently powered to detect a large effect size, the relatively small overall sample and predominance of female participants may limit the generalizability of findings. Future studies should aim for larger and more demographically diverse cohorts to improve external validity. Additional limitation was the short study duration of 21 days that may not be sufficient to capture all long-term metabolic and microbiome adaptations. While our short-term study provides valuable initial evidence of acute or early-phase responses in both oxidative stress markers and gut microbiome composition, we recognize the importance of longitudinal data to determine the persistence and clinical relevance of these effects, ideally through future interventions lasting several months to years. The latter is supported by the fact that while microbiome shifts were observed at the species level, overall diversity measures did not change significantly, indicating that more extended interventions may be necessary to achieve substantial microbial restructuring.

We also acknowledge that participants in this study were recruited based on their expressed interest in adopting a healthier lifestyle, which likely contributed to higher adherence and more favourable outcomes. This self-selection introduces a potential bias, as the findings may not be generalizable to less motivated individuals or clinical populations with lower baseline readiness for behaviour change.

## 5 Conclusion

This study highlights the benefits of dietary supplementation with whole food powders alongside the change to a healthier diet in reducing oxidative stress, modulating gut microbiota, and improving metabolic markers. The observed decrease in *Proteobacteria* and shifts in beneficial bacterial species suggest a healthier gut environment that may support long-term metabolic and immune health. Additionally, improvements in stress perception and kidney filtration efficiency underscore the broader physiological impact of whole food supplementation. However, the study’s limited duration and sample size indicate the need for longer trials to fully capture sustained microbiome and metabolic adaptations. These findings support the potential of targeted dietary interventions to facilitate the transition to healthier eating habits and improve well-being.

## Data Availability

The original contributions presented in the study are included in the article/Supplementary material, further inquiries can be directed to the corresponding author.

## References

[1] Koehler K Drenowatz C. Integrated role of nutrition and physical activity for lifelong health. Nutrients. (2019) 11:1437. doi: 10.3390/nu11071437, PMID: 31247924 PMC6682932

[2] Martínez Steele E Baraldi LG Louzada MLDC Moubarac J-C Mozaffarian D Monteiro CA. Ultra-processed foods and added sugars in the US diet: evidence from a nationally representative cross-sectional study. BMJ Open. (2016) 6:e009892. doi: 10.1136/bmjopen-2015-009892, PMID: 26962035 PMC4785287

[3] Guo W Pan B Sakkiah S Yavas G Ge W Zou W. Persistent organic pollutants in food: contamination sources, health effects and detection methods. Int J Environ Res Public Health. (2019) 16:4361. doi: 10.3390/ijerph16224361, PMID: 31717330 PMC6888492

[4] Bonakdar RA Sweeney M Dalhoumi S Adair V Garvey C Hodge T. Detoxification enhanced lifestyle intervention targeting Endotoxemia (DELITE) in the setting of obesity and pain: results of a pilot group intervention. Integr Med. (2020) 19:16–28. Available online at: https://pubmed.ncbi.nlm.nih.gov/33488302/PMC7815256 33488302

[5] Mostafalou S Abdollahi M. Pesticides and human chronic diseases: evidences, mechanisms, and perspectives. Toxicol Appl Pharmacol. (2013) 268:157–77. doi: 10.1016/j.taap.2013.01.025, PMID: 23402800

[6] Liu J Lewis G. Environmental toxicity and poor cognitive outcomes in children and adults. J Environ Health. (2014) 76:130–8. Available online at: https://www.neha.org/Images/resources/76.6-JEH-JanuaryFebruary-2014-Issue.pdf 24645424 PMC4247328

[7] Tuso PJ Ismail MH Ha BP Bartolotto C. Nutritional update for physicians: plant-based diets. Perm J. (2013) 17:61–6. doi: 10.7812/TPP/12-085, PMID: 23704846 PMC3662288

[8] Komarnytsky S Retchin S Vong CI Lila MA. Gains and losses of agricultural food production: implications for the twenty-first century. Annu Rev Food Sci Technol. (2022) 13:239–61. doi: 10.1146/annurev-food-082421-114831, PMID: 34813357

[9] Holt RR Schmitz HH Mhawish R Komarnytsky S Nguyen T Caveney PM. Comfort foods in the twenty-first century: friend or foe? Annu Rev Food Sci Technol. (2024) 16:433–58. doi: 10.1146/annurev-food-111523-122109 39661555

[10] Cooke NK Ash SL Goodell LS. Medical students’ perceived educational needs to prevent and treat childhood obesity. Educ Health (Abingdon). (2017) 30:156–62. doi: 10.4103/efh.EfH_57_16, PMID: 28928346

[11] Esquivel MK. Nutrition benefits and considerations for whole foods plant-based eating patterns. Am J Lifestyle Med. (2022) 16:284–90. doi: 10.1177/15598276221075992, PMID: 35706588 PMC9189583

[12] Dreher ML. Whole fruits and fruit Fiber emerging health effects. Nutrients. (2018) 10:1833. doi: 10.3390/nu10121833, PMID: 30487459 PMC6315720

[13] Komarnytsky S Wagner C Gutierrez J Shaw OM. Berries in microbiome-mediated gastrointestinal, metabolic, and immune health. Curr Nutr Rep. (2023) 12:151–66. doi: 10.1007/s13668-023-00449-0, PMID: 36738429

[14] Vong CI Rathinasabapathy T Moncada M Komarnytsky S. All polyphenols are not created equal: exploring the diversity of phenolic metabolites. J Agric Food Chem. (2022) 70:2077–91. doi: 10.1021/acs.jafc.1c07179, PMID: 35147422

[15] Elizabeth L Machado P Zinöcker M Baker P Lawrence M. Ultra-processed foods and health outcomes: a narrative review. Nutrients. (2020) 12:1955. doi: 10.3390/nu12071955 32630022 PMC7399967

[16] Panda C Komarnytsky S Fleming MN Marsh C Barron K Le Brun-Blashka S. Guided metabolic detoxification program supports phase II detoxification enzymes and antioxidant balance in healthy participants. Nutrients. (2023) 15:2209. doi: 10.3390/nu15092209, PMID: 37432335 PMC10181083

[17] Kumkum R Aston-Mourney K McNeill BA Hernández D Rivera LR. Bioavailability of anthocyanins: whole foods versus extracts. Nutrients. (2024) 16:1403. doi: 10.3390/nu16101403, PMID: 38794640 PMC11123854

[18] Karlsen MC Rogers G Miki A Lichtenstein AH Folta SC Economos CD. Theoretical food and nutrient composition of whole-food plant-based and vegan diets compared to current dietary recommendations. Nutrients. (2019) 11:625. doi: 10.3390/nu11030625, PMID: 30875784 PMC6471973

[19] Shi J Yu J Pohorly JE Kakuda Y. Polyphenolics in grape seeds-biochemistry and functionality. J Med Food. (2003) 6:291–9. doi: 10.1089/109662003772519831, PMID: 14977436

[20] Connolly EL Sim M Travica N Marx W Beasy G Lynch GS. Glucosinolates from cruciferous vegetables and their potential role in chronic disease: investigating the preclinical and clinical evidence. Front Pharmacol. (2021) 12:767975. doi: 10.3389/fphar.2021.767975, PMID: 34764875 PMC8575925

[21] Caliceti C Malaguti M Marracino L Barbalace MC Rizzo P Hrelia S. Agri-food waste from apple, pear, and sugar beet as a source of protective bioactive molecules for endothelial dysfunction and its major complications. Antioxidants. (2022) 11:1786. doi: 10.3390/antiox11091786, PMID: 36139860 PMC9495678

[22] Gómez-Zorita S González-Arceo M Fernández-Quintela A Eseberri I Trepiana J Portillo MP. Scientific evidence supporting the beneficial effects of Isoflavones on human health. Nutrients. (2020) 12:3853. doi: 10.3390/nu12123853, PMID: 33348600 PMC7766685

[23] Ha B. The power of plants: is a whole-foods, plant-based diet the answer to health, health care, and physician wellness? Perm J. (2019) 23:19–003. doi: 10.7812/TPP/19.003, PMID: 31496505 PMC6730944

[24] Martini D da Costa Ribeiro H Gately P Mattes R Re R Bier D. Positive nutrition: shifting the focus from nutrients to diet for a healthy lifestyle. Eat Weight Disord. (2023) 28:51. doi: 10.1007/s40519-023-01580-1, PMID: 37341796 PMC10284998

[25] Öhman L Bergdahl J Nyberg L Nilsson L-G. Longitudinal analysis of the relation between moderate long-term stress and health. Stress Health. (2007) 23:131–8. doi: 10.1002/smi.1130

[26] McMurdie PJ Holmes S. Phyloseq: an R package for reproducible interactive analysis and graphics of microbiome census data. PLoS One. (2013) 8:e61217. doi: 10.1371/journal.pone.0061217, PMID: 23630581 PMC3632530

[27] Shields-Cutler RR Al-Ghalith GA Yassour M Knights D. SplinectomeR enables group comparisons in longitudinal microbiome studies. Front Microbiol. (2018) 9. doi: 10.3389/fmicb.2018.00785, PMID: 29740416 PMC5924793

[28] Zhang X Yi N. NBZIMM: negative binomial and zero-inflated mixed models, with application to microbiome/metagenomics data analysis. BMC Bioinformatics. (2020) 21:488. doi: 10.1186/s12859-020-03803-z, PMID: 33126862 PMC7597071

[29] Hall KD Sacks G Chandramohan D Chow CC Wang YC Gortmaker SL. Quantification of the effect of energy imbalance on bodyweight. Lancet. (2011) 378:826–37. doi: 10.1016/S0140-6736(11)60812-X, PMID: 21872751 PMC3880593

[30] Milhem F Komarnytsky S. Progression to obesity: variations in patterns of metabolic fluxes, fat accumulation, and gastrointestinal responses. Meta. (2023) 13:1016. doi: 10.3390/metabo13091016, PMID: 37755296 PMC10535155

[31] Pagliai G Dinu M Madarena MP Bonaccio M Iacoviello L Sofi F. Consumption of ultra-processed foods and health status: a systematic review and meta-analysis. Br J Nutr. (2021) 125:308–18. doi: 10.1017/S0007114520002688, PMID: 32792031 PMC7844609

[32] Clarkson TW. Environmental contaminants in the food chain. Am J Clin Nutr. (1995) 61:682S–6S. doi: 10.1093/ajcn/61.3.682S, PMID: 7879738

[33] Fanzo J Rudie C Sigman I Grinspoon S Benton TG Brown ME. Sustainable food systems and nutrition in the 21st century: a report from the 22nd annual Harvard nutrition obesity symposium. Am J Clin Nutr. (2022) 115:18–33. doi: 10.1093/ajcn/nqab315, PMID: 34523669 PMC8755053

[34] Lee H-R Yoo S-J Kim J Park CK Kang SW. Reduction of oxidative stress in peripheral blood mononuclear cells attenuates the inflammatory response of fibroblast-like Synoviocytes in rheumatoid arthritis. Int J Mol Sci. (2021) 22:12411. doi: 10.3390/ijms222212411, PMID: 34830290 PMC8624216

[35] Weyand CM Shen Y Goronzy J. Redox-sensitive signaling in inflammatory T cells and in autoimmune disease. Free Radic Biol Med. (2018) 125:36–43. doi: 10.1016/j.freeradbiomed.2018.03.004, PMID: 29524605 PMC6128787

[36] Meulmeester FL Luo J Martens LG Mills K van Heemst D Noordam R. Antioxidant supplementation in oxidative stress-related diseases: what have we learned from studies on alpha-tocopherol? Antioxidants (Basel). (2022) 11:2322. doi: 10.3390/antiox11122322, PMID: 36552530 PMC9774512

[37] Zamek-Gliszczynski MJ Hoffmaster KA Nezasa K Tallman MN Brouwer KLR. Integration of hepatic drug transporters and phase II metabolizing enzymes: mechanisms of hepatic excretion of sulfate, glucuronide, and glutathione metabolites. Eur J Pharm Sci. (2006) 27:447–86. doi: 10.1016/j.ejps.2005.12.007, PMID: 16472997

[38] Aronica L Ordovas JM Volkov A Lamb JJ Stone PM Minich D. Genetic biomarkers of metabolic detoxification for personalized lifestyle medicine. Nutrients. (2022) 14:768. doi: 10.3390/nu14040768, PMID: 35215417 PMC8876337

[39] Moreira LDSG Fanton S Cardozo L Borges NA Combet E Shiels PG. Pink pressure: beetroot (*Beta vulgaris* rubra) as a possible novel medical therapy for chronic kidney disease. Nutr Rev. (2022) 80:1041–61. doi: 10.1093/nutrit/nuab074, PMID: 34613396

[40] Clare BA Conroy RS Spelman K. The diuretic effect in human subjects of an extract of *Taraxacum officinale* Folium over a single day. J Altern Complement Med. (2009) 15:929–34. doi: 10.1089/acm.2008.0152, PMID: 19678785 PMC3155102

[41] Deslippe AL Soanes A Bouchaud CC Beckenstein H Slim M Plourde H. Barriers and facilitators to diet, physical activity and lifestyle behavior intervention adherence: a qualitative systematic review of the literature. Int J Behav Nutr Phys Act. (2023) 20:14. doi: 10.1186/s12966-023-01424-2, PMID: 36782207 PMC9925368

[42] Rizzatti G Lopetuso LR Gibiino G Binda C Gasbarrini A. Proteobacteria: a common factor in human diseases. Biomed Res Int. (2017) 2017:1–7. doi: 10.1155/2017/9351507, PMID: 29230419 PMC5688358

[43] Shin N-R Whon TW Bae J-W. Proteobacteria: microbial signature of dysbiosis in gut microbiota. Trends Biotechnol. (2015) 33:496–503. doi: 10.1016/j.tibtech.2015.06.011, PMID: 26210164

[44] Vester-Andersen MK Mirsepasi-Lauridsen HC Prosberg MV Mortensen CO Träger C Skovsen K. Increased abundance of proteobacteria in aggressive Crohn’s disease seven years after diagnosis. Sci Rep. (2019) 9:13473. doi: 10.1038/s41598-019-49833-3, PMID: 31530835 PMC6748953

[45] Gupta V Sharma S Pal K Goyal P Agarwal D Chander J. Serratia, no longer an uncommon opportunistic pathogen - Case Series & Review of literature. Infect Disord Drug Targets. (2021) 21:e300821191666. doi: 10.2174/1871526521666210222125215, PMID: 33618650

[46] Coletto E Latousakis D Pontifex MG Crost EH Vaux L Perez Santamarina E. The role of the mucin-glycan foraging *Ruminococcus gnavus* in the communication between the gut and the brain. Gut Microbes. (2022) 14:2073784. doi: 10.1080/19490976.2022.2073784, PMID: 35579971 PMC9122312

[47] Kwoji ID Aiyegoro OA Okpeku M Adeleke MA. Multi-strain probiotics: synergy among isolates enhances biological activities. Biology. (2021) 10:322. doi: 10.3390/biology10040322, PMID: 33924344 PMC8070017

[48] Singh V Lee G Son H Koh H Kim ES Unno T. Butyrate producers, “the sentinel of gut”: their intestinal significance with and beyond butyrate, and prospective use as microbial therapeutics. Front Microbiol. (2023) 13:1103836. doi: 10.3389/fmicb.2022.1103836, PMID: 36713166 PMC9877435

