# Supplementary material for: Metabolic reset purification program improves antioxidant balance and gut microbiome in individuals transitioning to a healthier diet
Source: Front Nutr. 2025 Aug 11;12:1621709. doi: 10.3389/fnut.2025.1621709 (PMC12376428; doi:10.3389/fnut.2025.1621709)
Supplement: Supplementary file 1 [file Image_1.pdf]

Supplementary Material

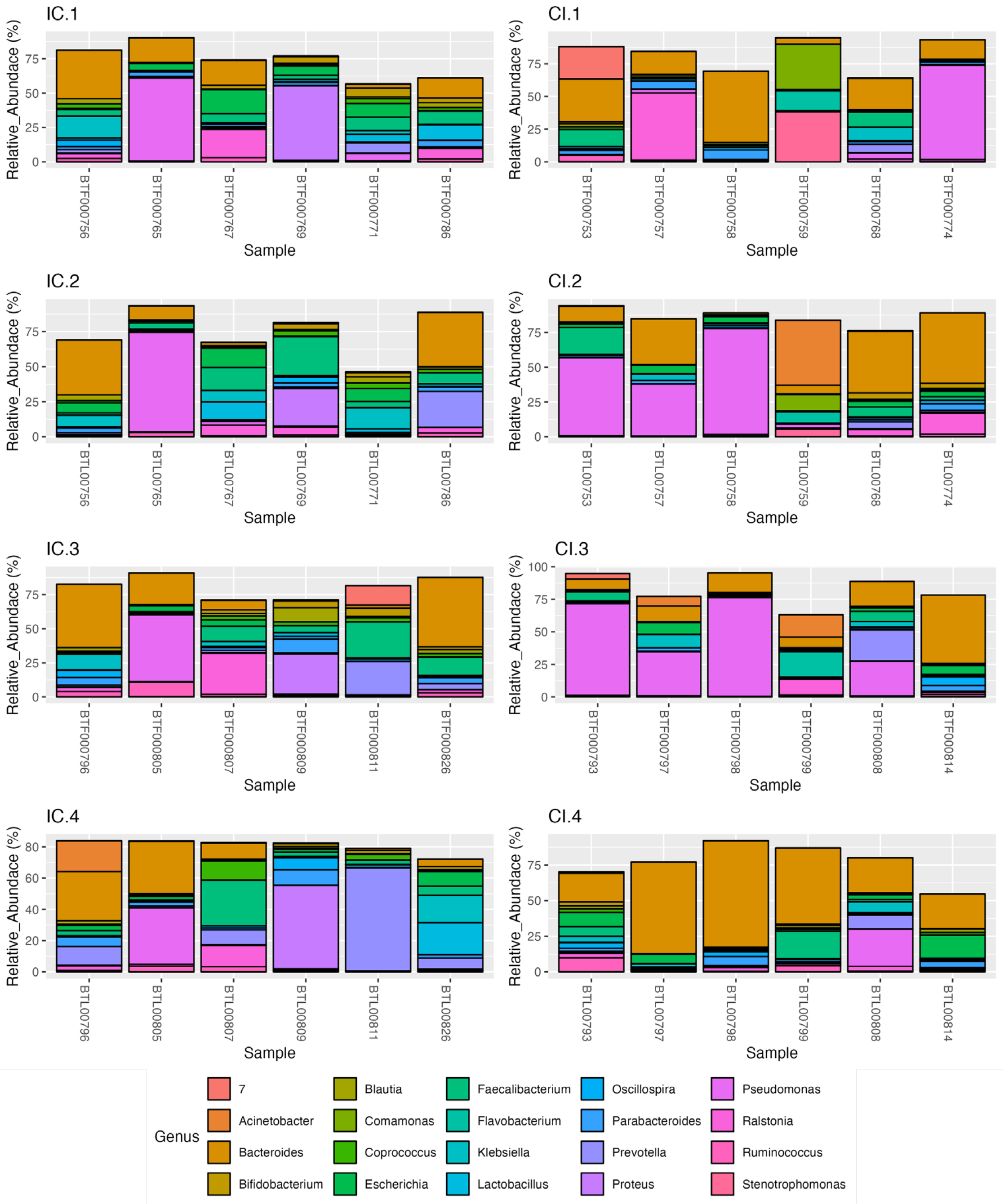

Supplementary Figure S1. Taxonomic distribution of top 20 bacterial genera in fecal microbiomes of individual participants following sequence 1 (CI) or sequence 2 (IC).

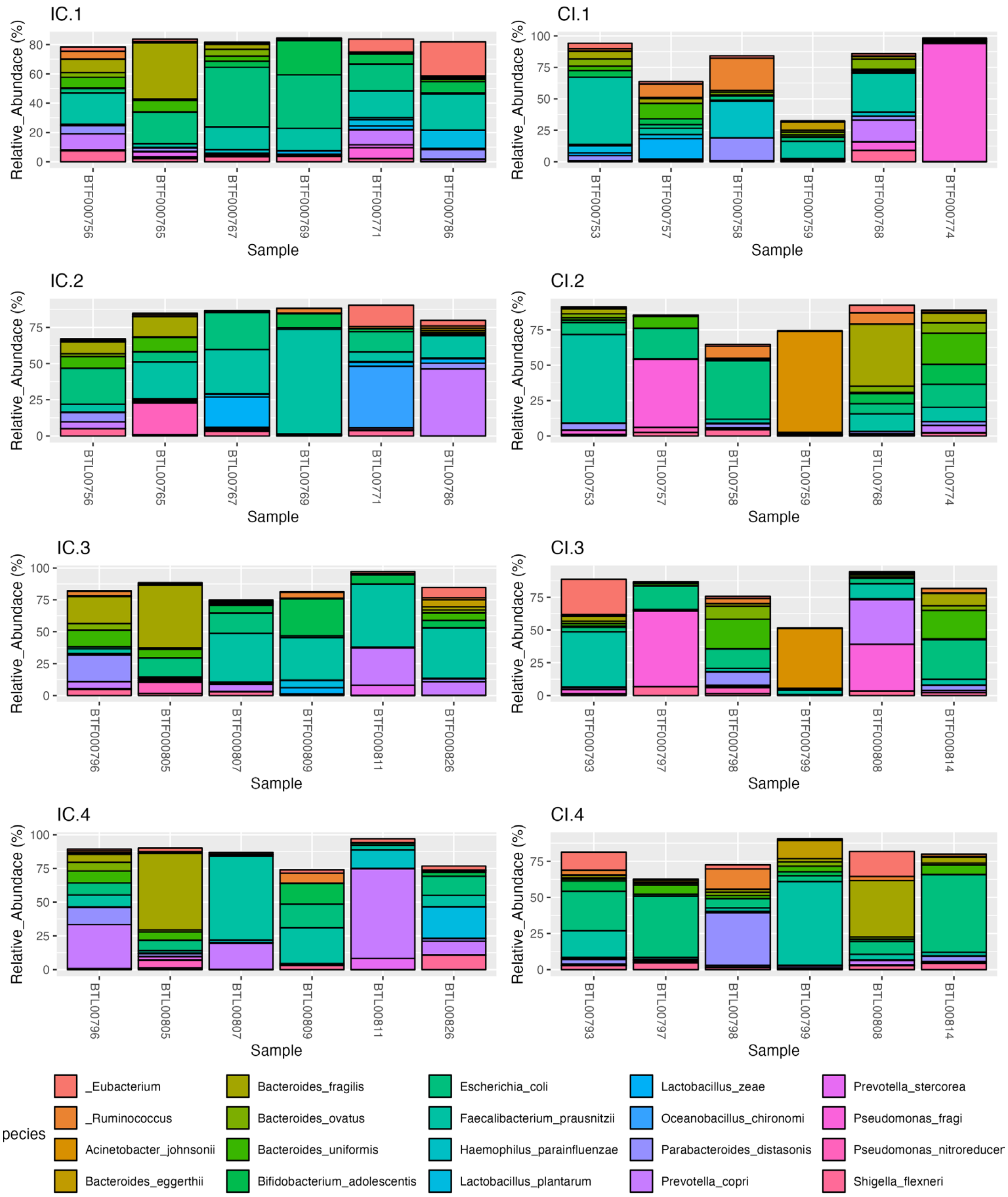

Supplementary Figure S2. Taxonomic distribution of top 20 bacterial species in fecal microbiomes of individual participants following sequence 1 (CI) or sequence 2 (IC).

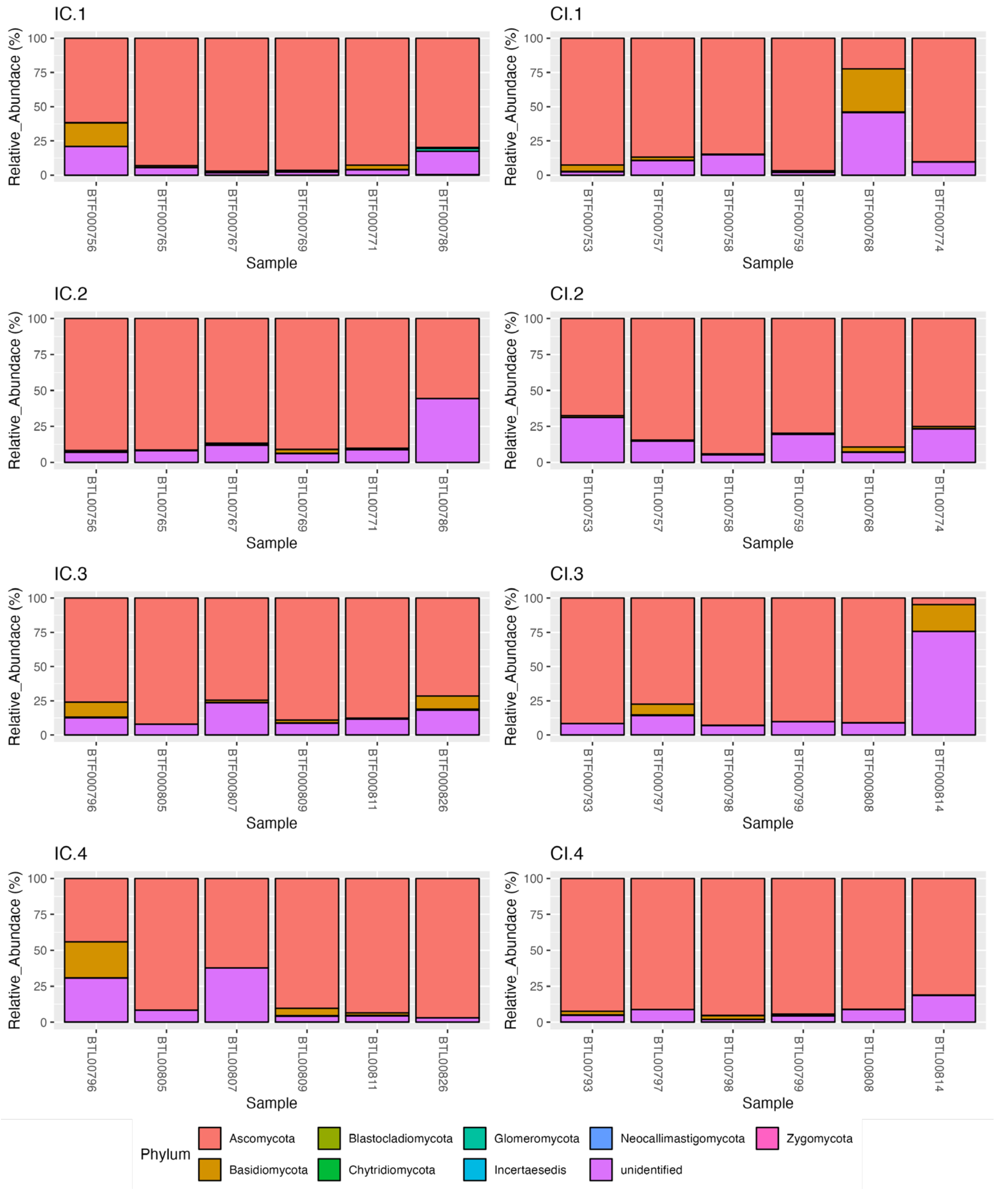

Supplementary Figure S3. Taxonomic distribution of top 9 fungal phyla in fecal microbiomes of individual participants following sequence 1 (CI) or sequence 2 (IC).

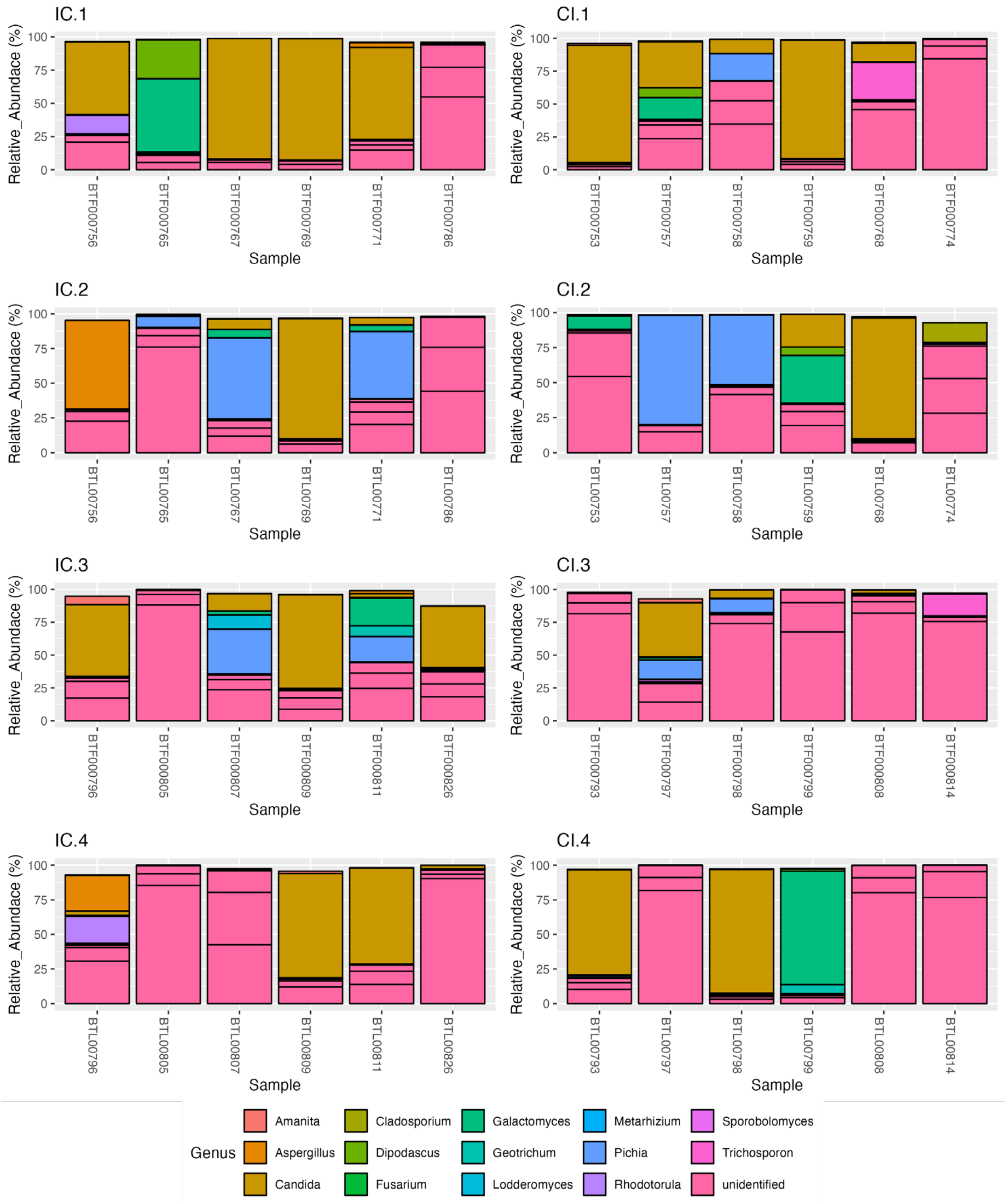

Supplementary Figure S4. Taxonomic distribution of top 15 fungal genera in fecal microbiomes of individual participants following sequence 1 (CI) or sequence 2 (IC).

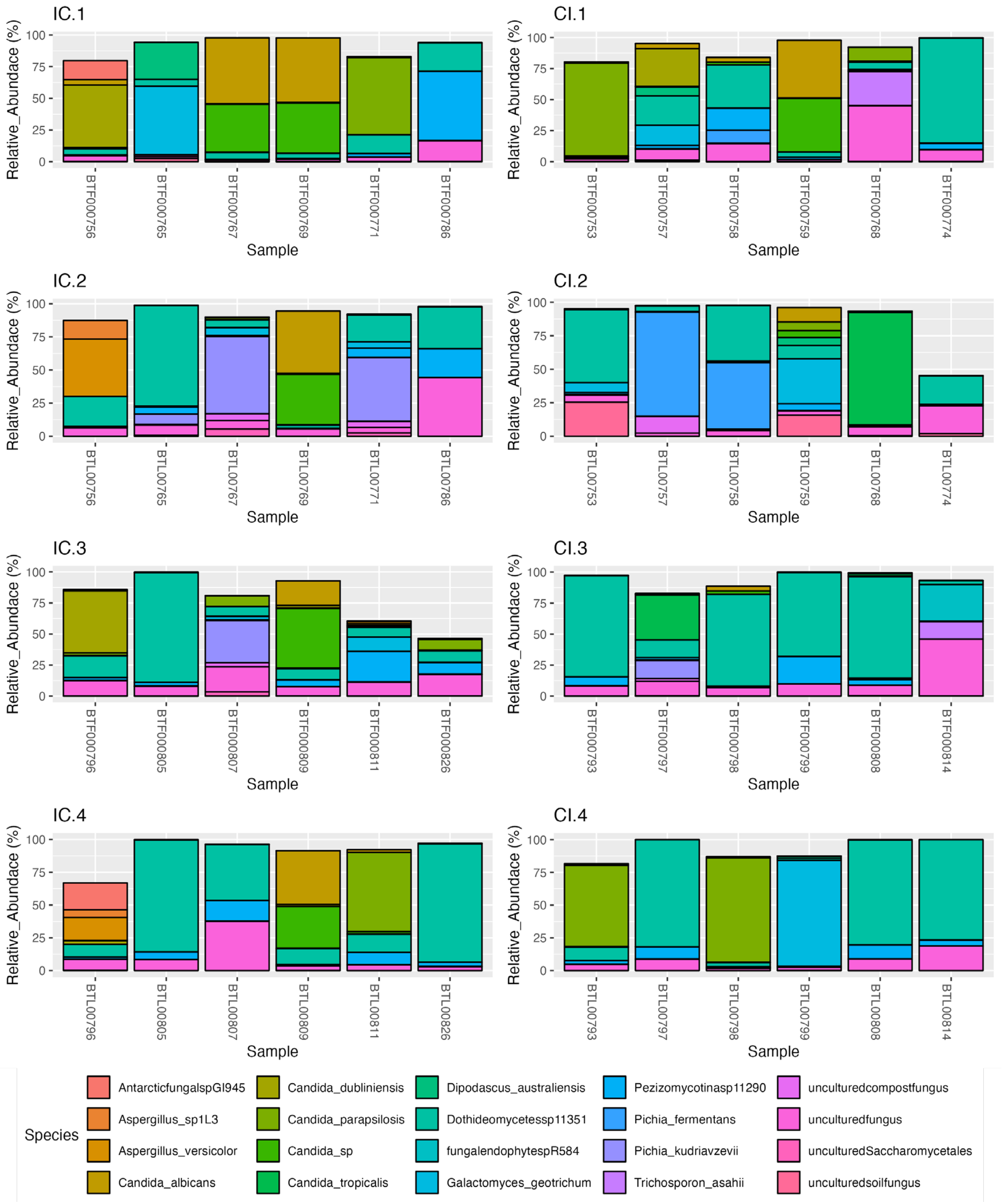

Supplementary Figure S5. Taxonomic distribution of top 20 fungal species in fecal microbiomes of individual participants following sequence 1 (CI) or sequence 2 (IC).

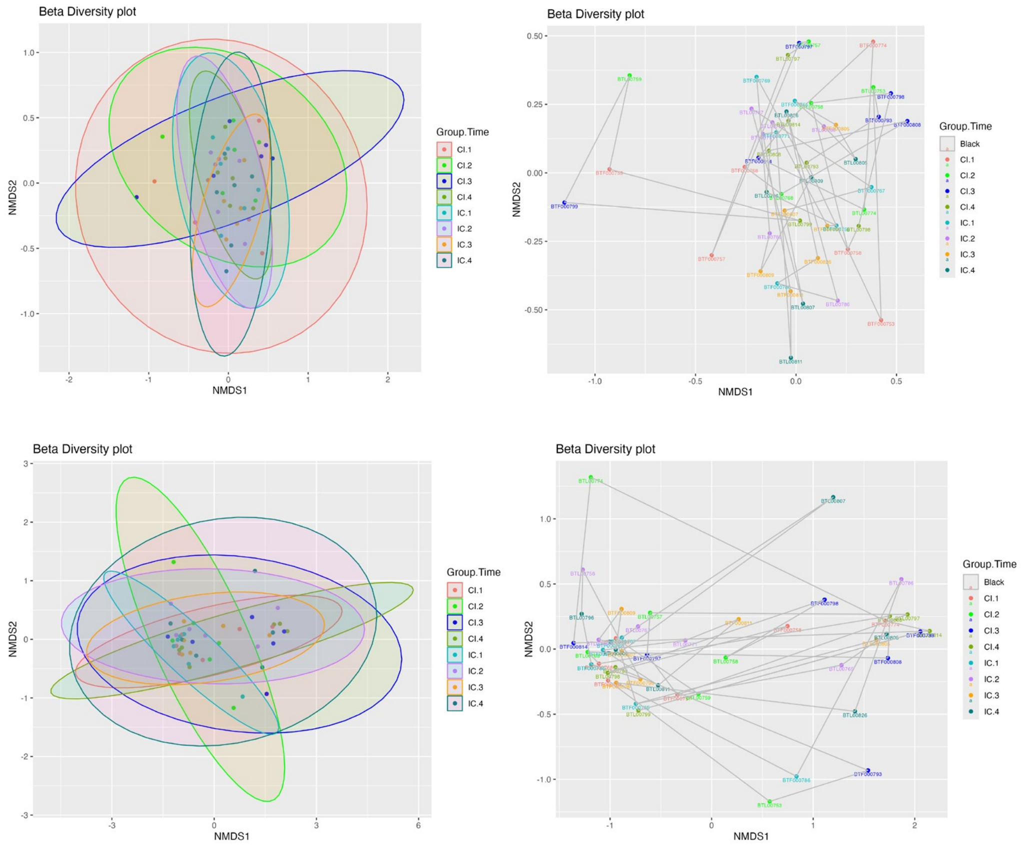

Supplementary Figure S6. Beta-diversity of bacterial (top) and fungal (bottom) microbiomes across all visits in control and intervention groups. Nonmetric multidimensional scaling (NMDS) using Bray-Curtis metrices showed no significant shifts ($p>0.05$).
